# NMR Spectroscopy of Human Eye Tissues: A New Insight into Ocular Biochemistry

**DOI:** 10.1155/2014/546192

**Published:** 2014-11-26

**Authors:** Tomasz Kryczka, Edward Wylęgała, Dariusz Dobrowolski, Anna Midelfart

**Affiliations:** ^1^Department of Neuroscience, Faculty of Medicine, Norwegian University of Science and Technology, Edvard Griegs Gate. 8, 3. Etg., 7489 Trondheim, Norway; ^2^Department of Experimental Pharmacology, Mossakowski Medical Research Centre, Polish Academy of Sciences, 02-106 Warsaw, Poland; ^3^Department of Ophthalmology, Medical University of Silesia, District Railway Hospital, 40-001 Katowice, Poland; ^4^Department of Ophthalmology, University Hospital, 7006 Trondheim, Norway

## Abstract

*Background*. The human eye is a complex organ whose anatomy and functions has been described very well to date. Unfortunately, the knowledge of the biochemistry and metabolic properties of eye tissues varies. Our objective was to reveal the biochemical differences between main tissue components of human eyes.* Methods*. Corneas, irises, ciliary bodies, lenses, and retinas were obtained from cadaver globes 0-1/2 hours postmortem of 6 male donors (age: 44–61 years). The metabolic profile of tissues was investigated with HR MAS ^1^H NMR spectroscopy.* Results*. A total of 29 metabolites were assigned in the NMR spectra of the eye tissues. Significant differences between tissues were revealed in contents of the most distant eye-tissues, while irises and ciliary bodies showed minimal biochemical differences. ATP, acetate, choline, glutamate, lactate, myoinositol, and taurine were identified as the primary biochemical compounds responsible for differentiation of the eye tissues.* Conclusions*. In this study we showed for the first time the results of the analysis of the main human eye tissues with NMR spectroscopy. The biochemical contents of the selected tissues seemed to correspond to their primary anatomical and functional attributes, the way of the delivery of the nutrients, and the location of the tissues in the eye.

## 1. Introduction

The human eye is a complex organ consisting of three main layers: the outermost layer composed of the cornea and sclera, the middle one that consists of the choroid, ciliary body, and the iris, and the innermost retina. Within these structures there are the vitreous body, the lens, and the aqueous humor. All of them differ with respect to their anatomy, function, location in the eye, metabolism, and in part the source of nutrients supply that is usually the blood plasma and/or the aqueous humor. They cooperate between themselves to provide the main functions of the eye: collecting light from the surrounding environment, regulating its intensity and focusing the images on the retina that are later converted into a set of electrical signals transmitted to the visual cortex [1].

The functions of the eye are often disturbed in severe eye diseases related to infections or injury or when diseases originate from genetic and/or environmental determinants. There is also a group of eye diseases that are partially related to some systemic diseases or disorders developing beyond the eye structures (for references see, e.g., [2, 3]). These systemic disorders seem to affect the homeostasis at the eye compartments, which is determined by a subtle balance between numerous factors. This balance is a resultant of the efficient operations of cells or tissues, regulating, for example, the chemical composition of the eye compartments, so as to maintain health and functioning of the organ, regardless of outer conditions or factors. Unfortunately, even though the anatomy and functions of eye tissues have been described very well to date, the knowledge about the between-tissues metabolic interactions based on their biochemical similarities or differences varies.

The aim of this study was to reveal the biochemical contents of the selected human eye tissues and relate them to the main anatomical and physiological features of these tissues.

For this purpose we have assumed that the selected anatomical structures of the eye, albeit consisting of many cell types, form independent anatomical and functional entities. The main function (or functions) of every single anatomical entity has been a resultant of all metabolic or biochemical processes, enzymatic and nonenzymatic ones, running in various cells and noncellular compartments of the selected tissue. These entities cooperate between themselves to maintain the proper functions of the eye. In this “holistic approach” the functions of so defined structures should be related to the composition of the free low molecular weight compounds in the selected tissues/entities.

To fulfill the goal of this study we have used nuclear magnetic resonance (NMR) spectroscopy; the analytical technique that allows obtaining large amounts of biochemical data from a single sample. To identify the most meaningful biochemical compounds accounting for the largest possible variance in the data sets of the investigated tissues we have used the principal component analysis (PCA) approach. This method allows correlating the influence of biochemical compounds (determined as principal components) on the differentiation pattern of the eye tissues in respect to their biochemistry. The PCA has been completed with the statistical comparison of the levels of the individual biochemical compounds between the investigated tissues.

## 2. Methods

The study adhered to the tenets of the Declaration of Helsinki and local regulations with regard to the human studies and the use of human tissues and organs and has been filed with the Bioethics Committee of the Medical University of Warsaw, which has acknowledged the protocol of the study and has raised no objection.

### 2.1. Eye Tissues

Cadaver globes eyes were obtained 0-1/2 hours postmortem from 6 male donors (age: 44–61 years). The procedure was performed at the hospital in Poland on patients with confirmed brain death, which resulted from a former traumatic cerebrovascular damage. Then, corneas, iris, ciliary bodies, lenses, and retinas were separated from the eye globes and instantly frozen and stored at −80°C. No systemic or chronic disease or recent medication was reported in donors. For the further research purposes the samples were transported to Norway on dry ice. Prior to the analysis, the respective frozen tissues were quickly cut by hand into 2 × 2 × 1 mm pieces, weighted, and introduced to the HR-MAS rotor.

### 2.2. HR MAS ^1^H NMR Spectroscopy

HR MAS ^1^H NMR (high-resolution magic angle spinning proton nuclear magnetic resonance) spectroscopy was performed on a Bruker Avance DRX600 spectrometer (14.1 T, Bruker BioSpin GmbH, Rheinstetten, Germany) operating at 600.132 MHz for protons. Tissue samples were immersed in D_2_O in a zirconia 4 mm diameter (50 *μ*L) HR-MAS rotor. Sodium [2,2,3,3-d_4_]3′-trimethylsilylpropionate (d_4_-TSP, 25 mM) was used as an internal shift reference. The spectra were recorded at 4°C using a 4 mm ^1^H/^13^C MAS probe. The samples were spun at 5000 Hz and the number of scans was 512. Water suppression was done employing a presaturation selective pulse. Exponential line broadening of 0.3 Hz was used. Carr-Purcell-Meiboom-Gill (CPMG) spectra analysis was conducted using special software for the analysis of complex mixtures (MestreNova 5.1.0, Mestrelab Research, Spain). Peak areas were measured using absolute integrals and were normalised by the wet weight of the samples (weight range of corneas: 7.3–12.3 mg; ciliary bodies: 7.5–12.4 mg; iris: 7.6–12.1 mg; lens: 7.4–12.5 mg; retinas: 7.2–12.3 mg) and assignment of the metabolites in the spectra were performed as described earlier [4].

### 2.3. Statistical Analysis

^1^H NMR spectral data were normalized by sample wet weight and transferred into Unscrambler software (CAMO, Oslo, Norway) for PCA processing and then to statistical package R (see: http://www.r-project.org/). The number of principal components to include in the cross-validation analysis was assessed by the leverage correction validation method as the number of components that gave the minimal total validation X-variance. Next, the cross-validation method with mean centering was employed. The analytical procedure was repeated for the peak integrals of the biochemical compounds identified in the NMR spectra. To reveal specific grouping and the relationship between samples, the score plot of the first principal component (PC1) versus the second principal component (PC2) was interpreted.

To compare individual components from NMR spectra of investigated tissues, peak integrals were measured and normalized by sample wet weight. The assumptions of normality and homogeneity of variances of groups of samples (ciliary body, cornea, iris, lens, and retina) were checked by Shapiro-Wilk and Levene tests, respectively. If both these assumptions were met, the one-way ANOVA followed by Tukey's HSD test for multiple comparisons (with an adjustment for mild unequal sample sizes) were used. When normality was met but homogeneity was not met, the ANOVA with method of Welch for treating unequal variances was applied and multiple comparisons by *t*-tests with Holm's method for *P* value assessment were performed. In opposite situation the Kruskal-Wallis test followed by pairwise Wilcoxon rank sum test (with Holm's method for *P* value assessment) were performed. All statistical tests were two-sided and the results were considered statistically significant if *P* value was <0.05.

## 3. Results

**Figure 1:**
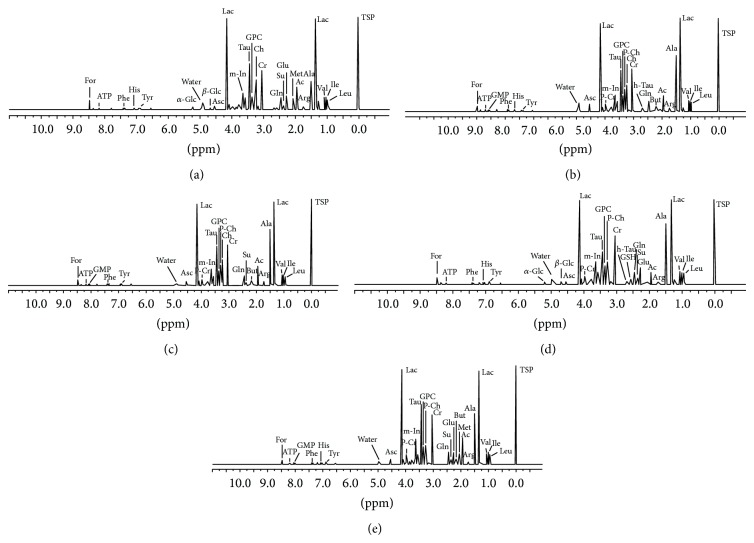
Representative HR MAS ^1^H NMR spectra of the intact human eye tissues. (a) cornea, (b) ciliary body, (c) iris, (d) lens, and (e) retina. The ppm values were assigned using sodium [2,2,3,3-d_4_]3′-trimethylsilylpropionate (TSP) as the reference substance at 0 ppm. Ac: acetate; Ala: alanine; Arg: arginine; Asc: ascorbate; ATP: adenosine triphosphate; But: butyrate; Ch: choline; Cr: creatine; For: formate; *α* *-*Glc: *α*-glucose; *β* *-*Glc: *β*-glucose; Glu: glutamate; Gln: glutamine; GMP: guanosine monophosphate; GPC: glycerophosphocholine; GSH: glutathione; His: histidine; h-Tau: hypo-taurine; Ile: isoleucine; Lac: lactate; Leu: leucine; m-In: myo-inositol; Met: methionine; Phe: phenylalanine; P-Ch: phosphocholine; P-Cr: phosphocreatine; Su: succinate; Tau: taurine; Tyr: tyrosine; Val: valine; ppm: parts per million.

A total of 29 metabolites were assigned in the ^1^H NMR spectra of eye tissues within the region from 0.5 to 10 ppm based on their characteristic chemical shifts and multiplicities. There were 23 compounds assigned in the corneal spectra, 22 compounds in the iris spectra, and 25 compounds in the spectra of each of the following tissues: the ciliary bodies, lens, and retinas (Figures 1(a)–1(e)).

### 3.1. PCA Results

**Figure 2:**
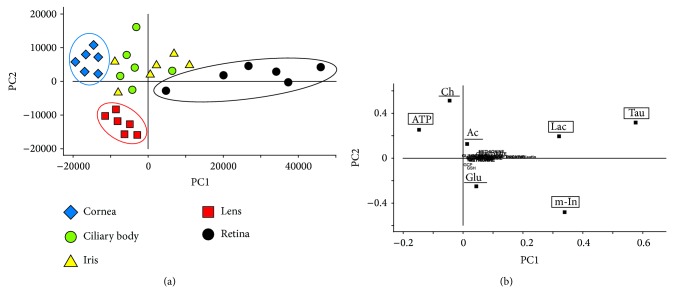
Principal component analysis of human eye tissues. (a) Score plot of the 1st versus the 2nd principal component (PC1 versus PC2) of ^1^H NMR spectra. The groupings of the samples were marked with colorful ellipses. (b) Loading profile of the principal components of the ^1^H NMR spectra of the eye tissues. Metabolites described in lower cases in the loading profile indicate negligible influence of these compounds on the grouping pattern. The biochemical compounds that served as PC1 were marked with the square frames. The biochemical compounds that served as PC2 were underlined. For metabolite labels see legend for Figure 1.

The score plots of PC1 versus PC2, whose components accounted for 64% and 13%, respectively, of the total variation in the NMR spectra of the samples, showed considerable dispersion of the samples along the PC1 and PC2 axes (Figure 2(a)). The shift along the PC1 axis was a result of alterations in the content of several metabolites (Figure 2(b)). A low PC1 score was associated with the high ATP content, while a high PC1 score was related mainly, to high contents of lactate, myoinositol, and taurine (Figure 2(b)). Corneal samples showed a shift toward lower PC1 score, while retinal ones were shifted along the high scores of PC1 (Figure 2(a)). The remaining tissue samples were separated along the PC2 axis, in which the high PC2 scores were mainly related to low choline and acetate contents in the tissue, and low PC2 scores were associated mostly with high levels of glutamate in the samples (Figure 2(b)). PC2 allowed a distinct separation of lens samples from the remaining ones (Figure 2(a)). Finally, PCA revealed significant biochemical differences between the cornea, lens, retina, and the group of ciliary body and the iris samples.

### 3.2. Amino Acids

**Figure 3:**
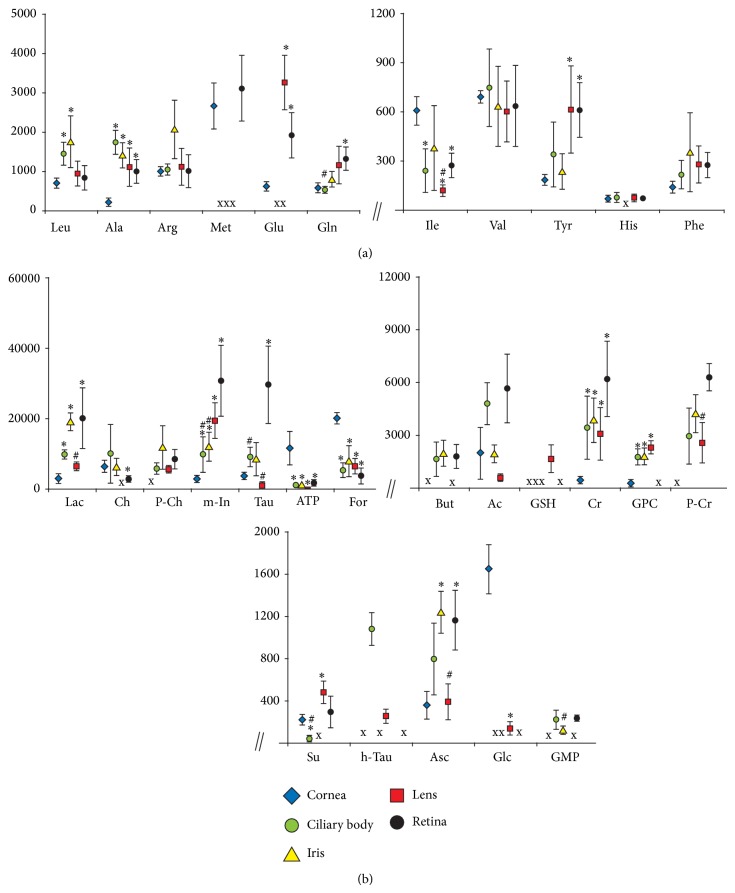
The contents of assigned biochemical compounds in various eye tissues. (a) amino acids; (b) nonamino acid compounds. Data are means ± SEM of HR MAS ^1^H NMR peak integrals divided by tissue wet weight; ^*^ *P* < 0.05 versus the respective cornea group value; ^#^ *P* < 0.05 versus the respective retina group value; X—no detectable metabolite peak in the respective NMR spectra. For metabolite labels see legend for Figure 1.

Statistical analysis of the levels of the amino acids in the samples revealed significant differences between the eye tissues (Figure 3(a), Table 1). The difference between the cornea and the ciliary body was related to the contents of leucine, alanine, glutamine, and isoleucine. Similarly, the difference between the cornea and the iris was reflected in the levels of leucine and alanine, between the cornea and the lens in the levels of alanine, isoleucine, and tyrosine and between the cornea and the retina in the amounts of alanine, glutamine, isoleucine, and tyrosine. In addition, retinas differed significantly from ciliary bodies and lenses in the content of glutamate and isoleucine, respectively. The levels of some of the metabolites were below the detection threshold: methionine was not assigned in the ciliary body, iris, and lens samples. Similarly, glutamate was not assigned in the ciliary bodies and the irises and histidine in the irises (Figure 3(a)).

### 3.3. Nonamino Acid Low Molecular Weight Metabolites

Statistical analysis of the levels of the nonamino acid low molecular weight metabolites revealed significant differences between various eye tissues. Corneas compared to ciliary bodies showed higher levels of ATP, formate, and succinate, and lower levels of creatine, glycerophosphocholine (GPC), lactate, and myoinositol. Higher levels of ATP and formate and lower levels of ascorbate, creatine, GPC, lactate, and myoinositol appeared in corneas compared to irises. Corneas compared to lenses showed higher levels of ATP, formate, and glucose and lower levels of creatine, GPC, myoinositol, and succinate. Higher levels of ATP, formate, and choline and lower levels of ascorbate, creatine, lactate, myoinositol, and taurine appeared in corneas compared to retinas (Figure 3(b), Table 1).

In addition, retinas differed significantly from ciliary bodies and irises in the levels of succinate and guanosine monophosphate (GMP), respectively. Myoinositol levels in both the ciliary bodies and the irises were lower than in the retinas and the same pattern was observed in the levels of taurine in the ciliary bodies and the lenses. Ascorbate, lactate, and phosphocreatine were significantly reduced in lens samples in comparison to retinas (Figure 3(b)). The levels of some of the metabolites were below the detection threshold: hypotaurine, phosphocholine and phosphocreatine were not assigned in the corneas. Succinate and choline were below the detection threshold in irises and lenses, respectively.

A few compounds were shown to be undetectable in more than one tissue: glucose, in the ciliary bodies, irises and retinas; hypotaurine, in the corneas, irises, and retinas, while butyrate and GMP, in the corneas and lenses. Glutathione was assigned only in the lens samples (Figure 3(b)).

## 4. Discussion

NMR spectroscopy is a research technique that exploits the magnetic properties of certain atomic nuclei, for example, protons. It determines the physical and chemical properties of the molecules in which they are contained. It relies on the phenomenon of nuclear magnetic resonance and can provide detailed information about the structure, dynamics, reaction state, and chemical environment of the molecules. The intramolecular magnetic field around an atom in a molecule changes the resonance frequency, thus giving access to details of the electronic structure of a molecule and then allowing identification of the biochemical compound. The limitations on the strength and inherent inhomogeneity of the magnetic fields restrict the* in vivo* studies to a handful of high-concentration metabolites.* Ex vivo* NMR spectroscopy of the excised tissue generate spectra that enable separation and relative quantification of the majority of metabolites in the tissue [5, 6].

The significant improvements in the quality of spectra of tissues appeared when magic angle spinning (MAS), allowing analysis of intact samples and high resolution (HR) techniques, were first applied in NMR spectroscopy. Due to the molecular constraints of semisolids, ordinary high resolution (HR) NMR spectroscopy of the excised tissue resulted in broadening of the metabolite peaks in the NMR spectra. As dipole couplings and chemical shift anisotropy both are scaled according to the term (3cos⁡^2^⁡*θ* − 1), positioning the sample at the so-called magic angle of 54.7° to the magnetic field and then spinning it rapidly about its own axis reduced these interactions. With this technique (HR MAS) resonances from lipids and macromolecules in tissues were attenuated and a whole range of low molecular substances could be assigned in the spectra [5].

NMR spectroscopy does not require a complicated sample preparation and is nondestructive and relatively rapid. The investigated sample is only a subject of deep freezing between collecting and introducing of the frozen sample to the NMR instrument for measurements. As an extraction is not necessary prior to the analysis with HR MAS NMR spectroscopy, possible negative effects of this procedure, particularly on sensitive metabolites, were avoided. Hence, the biochemical contents of such frozen samples reflected the respective* in vivo* values [5, 7].

The blood supply is considered to be the optimal way of the nutrients' delivery and waste products' removal in any organ, including the eye and it significantly limits the predisposition of the cells or tissues to a spontaneous dysfunction or damage [8, 9]. On the other hand, some chronic systemic diseases might lead to an increase of toxic metabolites in the blood plasma, which also might, for example, affect and damage the retina. One example is diabetes; when nontoxic compound, glucose, exceeds its normal levels, it causes metabolic changes, which in the long-term observation period influences the functionality of the retina. In addition, even the dietary mistakes may cause irreversible damage; the most extreme example is an accidental consumption of methanol [2, 3, 10].

Another source of nutrients in the eye is an aqueous humor, which is a clear fluid derived from the blood plasma. Free amino acid levels in the aqueous humor are nearly equivalent to those found in the blood plasma, but the protein content of the aqueous humor in the anterior chamber is less than 1% of that found in the plasma [11–15]. This is to ensure that potential antigens in the bloodstream are prevented from reaching the eye tissues as proteins create turbidity and the turbidity scatters light, which degrades the optical efficiency of the eye.

The cornea, due to its location, is considered the most outer part of the eye and the main physical barrier to external factors with a high metabolic activity in the epithelium and the endothelium [16–20]. Oxygen and some important nutrients can reach the cornea from the tear film, limbal blood vessels, and aqueous humor, though the aqueous humor is the main nutritive source [12, 15, 21, 22]. The paper by Redbrake et al. [23] has revealed that despite the lack of regular blood vessels supply, the corneal biochemistry is not neutral to the metabolic changes related to chronic systemic diseases or causes of death. This observation has been recently confirmed with the study showing that the corneas of patients with liver cirrhosis or cardiovascular diseases differ significantly in biochemical content from the corneas obtained from healthy donors [24].

In contrast to the cornea, the retina is unique in having the highest oxygen consumption per unit weight of any tissue in the human body and in having two separate circulatory systems to meet this metabolic demand [8, 9, 25]. PCA revealed some biochemical differences between the vascular (ciliary body, iris, and retina) and the avascular eye tissues (cornea and lens), but the most characteristic were revealed for the most distant structures, the cornea and the retina (Figure 2(a)). ATP was identified as the one of PC1 compounds responsible for the distant grouping of cornea samples in the score plot (Figure 2). The possible explanation of high levels of ATP in the corneas comparing to the remaining tissues (Figure 3(b)) could be that the protection of the eye against the environmental factors and maintaining the integrity of the corneal tissue require the additional energy reserves in cells [1, 16, 18, 26–29].

The levels of remaining PC1-score compounds (lactate, myoinositol, and taurine) were significantly lower in the cornea samples than in the retina ones (Figure 3(b)). Both, myoinositol and taurine can be classified as the main osmolytes. However, myoinositol may further function as a cellular signal transducer and has a significant role in growth and differentiation, while taurine has been additionally thought to enhance cell survival as a membrane stabilizer or an antioxidant [30–32]. Considering that a significant portion of the potentially damaging factors influencing the human eye are the free radical species, the increased metabolic turnover of these compounds in corneas aiming to protect the organ from damage could explain the differences revealed in the statistical analyses (Figures 2 and 3).

Interestingly, the retina contains dozens of various cell types and has an especially high demand for energy [9, 25], but the measurements with NMR spectroscopy have showed significantly lower levels of ATP in the tissue than in the cornea (Figure 3(b)). Possibly the high metabolic turnover rate of ATP related to the energy demand decreased the levels of this metabolite in retina. However, the relatively high concentrations of the lactate in the tissue (Figure 3(b)) suggest that the production of energy is after all maintained. The lactate could be produced from the pyruvate that was created from glucose. In such a biochemical pathway NAD+ is regenerated [33, 34] insuring the energy stability in retina cells.

Based on PC2-score, consisting of acetate, choline, and glutamate, a further separation of the tissues has been observed, whereas ciliary body and the iris remain one group of samples in the score plot (Figure 2(a)). Both, the iris and the ciliary body, despite being different anatomical and functional structures, are supplied from the same major arterial circle located in the ciliary body [1, 8, 11] that could minimize the biochemical differences between both these tissues. In contrast to them, the avascular lens depends only on the aqueous humor as the main nutritive source [1, 12, 35]. Choline (one of the membrane phospholipids' precursors [36, 37]) has not been assigned in the lens (Figures 1(d) and 3(b)), while the acetate level was significantly lower in the tissue, comparing to other eye tissues (the statistical significance not shown in the figure). The low levels or below the detection threshold levels of both compounds might suggest a better stability of the cellular membranes in the lens than in any of the other investigated eye tissues. This thesis could be supported with the measurements of butyrate: it has not been assigned in the lens, neither in the corneal samples (Figures 1(a), 1(d) and 3(b)). This short chain fatty acid usually comes from the degraded long-chain fatty acids and it is known to induce apoptosis when the intracellular concentration of this compound exceeds an appropriate level [38]. As fatty acids are considered among main components of cell membranes, the absence (or low concentration) of butyrate might indicate solid antiapoptotic or antinecrotic properties of cells and tissue, while the reverse would suggest an increased degradation of cell structures, including cell membranes.

Glutathione is the powerful antioxidant produced mainly in the lens epithelium from the interactions between glutamate, cysteine, and glycine in the cells [39, 40]. High levels of glutathione assigned in lens (Figures 1(d) and 3(b)) should minimize the influence of free radical species prevalent in the aqueous humor (or generated by UV) on the lens. In addition, low levels of isoleucine and undetectable concentrations of methionine (Figures 1 and 3(a)) suggest a high consumption rate of these amino acids for synthesis of crystallins that compose over 90% of the proteins within the lens [41]. Meanwhile high levels of glutamate (Figure 3(a)) could be linked with a high glutathione production in lens [39, 40]. Interestingly, glutamate was also revealed in corneas and retinas (Figures 1(a), 1(e) and 3(a)) with no detectable levels of glutathione (Figures 1 and 3(b)) that could indicate the extraordinary antioxidative activities in both tissues.

Importantly, an absence of the biochemical compound in the NMR spectra did not mean that the investigated tissue did not contain it at all. On the contrary, some specific types of cells might contain high concentrations of the biochemical compound in the cytoplasm, while the marginal volume of these cells in the investigated tissue homogenate makes it invisible for the analytical instrument. A good example is glutathione occurring in high concentrations in some retina cells (e.g., Müller cells [42]) that have not been assigned in the tissue homogenate with NMR spectroscopy (Figures 1 and 3(b)).

The biochemical properties of the eye tissues and their grouping pattern, vascular versus avascular ones according to their biochemical profiles (Figure 2), raise the questions about the correlation between the contents of the blood plasma or aqueous humor and the biochemical composition of the main eye tissues. In theory, considering that some chronic systemic diseases might influence the contents of the blood plasma, such biochemical abnormalities could be transferred to the eye tissues via nutritional pathways. If this is the case, the question appears of how “severe” the biochemical abnormalities in the blood plasma should be or how long they should last to initiate any biochemical changes in the eye tissues. Then, how “severe” should these changes be in the eye tissues to initiate any eye disease? The results of this study have not answered these questions; however, considering the previous reports showing the biochemical differences in corneas obtained from various disease-burdened donors [24], it seems reasonable to combine the analysis of the blood plasma and eye tissues with the metabolomics approach.

## 5. Conclusions

Summarizing, the HR MAS ^1^H NMR spectroscopy allowed assigning a number of free low molecular weight biochemical compounds in the main eye tissues of healthy donors. The biochemical contents of these tissues seemed to correspond to their primary anatomical and functional attributes. The most significant biochemical differences were revealed for the tissues that differed mainly in both the way of delivery of the nutrients and the location of the tissues in the eye. The prospect of the analysis of human eye tissues with the metabolomics methods like NMR spectroscopy opens new perspectives of scientific research on the development of eye diseases.

## Figures and Tables

**Table 1:** Statistical significance of biochemical differences between the eye tissues.

Metabolite	Homogeneity	Normality	Type of analysis	Cornea-CB^***^	Cornea-iris^***^	Cornea-lens^***^	Cornea-retina^***^	CB-retina^***^	Iris-retina^***^	Lens-retina^***^
Ac	−^*^	+^**^	AW	−	−	−	−	−	−	−
Ala	+^*^	−^**^	KW	+	+	+	+	−	−	−
Arg	−	+	AW	−	−	−	−	−	−	−
Asc	+	+	A	+	+	−	+	−	−	+
ATP	−	+	AW	+	+	+	+	−	−	−
But	+	+	A	n/a	n/a	n/a	n/a	−	−	n/a
Ch	−	+	AW	−	−	n/a	+	−	−	n/a
Cr	+	−	KW	+	+	+	+	−	−	−
For	−	+	AW	+	+	+	+	−	−	−
GPC	+	+	A	+	+	+	n/a	n/a	n/a	n/a
Gln	−	+	AW	+	−	−	+	+	−	−
Glc	−	+	AW	n/a	n/a	+	n/a	n/a	n/a	n/a
Glu	−	+	AW	n/a	n/a	+	+	n/a	n/a	−
GMP	−	+	AW	n/a	n/a	n/a	n/a	−	+	n/a
GSH	−	−	−	n/a	n/a	n/a	n/a	n/a	n/a	n/a
His	+	+	A	−	n/a	−	n/a	n/a	n/a	n/a
h-Tau	+	+	A	n/a	n/a	n/a	n/a	n/a	n/a	n/a
Ile	+	−	KW	+	−	+	+	−	−	+
Lac	−	+	AW	+	+	−	+	−	−	+
Leu	−	+	AW	+	+	−	−	−	−	−
Met	+	+	A	n/a	n/a	n/a	−	n/a	n/a	n/a
m-In	−	+	AW	+	+	+	+	+	+	−
P-Ch	−	+	AW	n/a	n/a	n/a	n/a	−	−	−
P-Cr	+	+	A	n/a	n/a	n/a	n/a	−	−	+
Phe	+	+	A	−	−	−	−	−	−	−
Su	+	−	KW	+	n/a	+	−	+	n/a	−
Tau	−	+	AW	−	−	−	+	+	−	+
Tyr	−	+	AW	−	−	+	−	−	−	−
Val	+	+	A	−	−	−	−	−	−	−

^*^homogeneity of the groups (+/−, yes/no); ^**^normality of the distribution within the groups (+/−, yes/no); ^***^statistical significance of differences between the groups of samples (+/−, positive/negative); A: one-way analysis of variance (ANOVA); AW: ANOVA with method of Welch for treating unequal variances; K: the Kruskal-Wallis test; n/a: not applicable due to the lack of data for one or both groups of samples; CB: ciliary body. For metabolite labels see legend for Figure 1.
